# Supplementary material for: Research Trends and Methodological Approaches of the Impacts of Windstorms on Forests in Tropical, Subtropical, and Temperate Zones: Where Are We Now and How Should Research Move Forward?
Source: Plants (Basel). 2020 Dec 4;9(12):1709. doi: 10.3390/plants9121709 (PMC7762080; doi:10.3390/plants9121709)
Supplement: Supplementary file 1 [file plants-09-01709-s001.pdf]

Supplementary Materials

Table S1. Sample main matrix used in data categorization. The subsequent data categorization and analysis was based on this main matrix.

Sequence No.	File Name	Journal name	Forest type (TRF, SUF, TEF)	Study site	Coordinates		Windstorm	Study duration		Major effect				Methodological approaches (observational or controlled)	Type of plot (permanent or temporary)	Other notes
					LAT	LONG		Start	End	MCI	SP	CE	L			

Abbreviations: LAT- Latitude; LONG- Longitude; MCI-molecullar, cellular, individual level; SP-Species, population level; CE- community, ecosystem level.
